# Prevalence of Spontaneous Bacterial Peritonitis (SBP) in Hepatitis B (HBV), and Hepatitis C (HCV) Liver Cirrhosis: A Systematic Review and Meta-Analysis

**DOI:** 10.3390/healthcare11020275

**Published:** 2023-01-16

**Authors:** Kizito Eneye Bello, Ahmad Adebayo Irekeola, Sameer Badri Al-Mhanna, Okolo Martin-Luther Oseni, Adejo Patience Omebije, Rafidah Hanim Shueb, Nazri Mustaffa

**Affiliations:** 1 Department of Medical Microbiology and Parasitology, School of Medical Sciences, Universiti Sains Malaysia, Health Campus, Kubang Kerian 16150, Kelantan, Malaysia; 2 Department of Microbiology, Faculty of Natural Science, Kogi State University (Prince Abubakar Audu University), Anyigba 1008, Kogi State, Nigeria; 3 Microbiology Unit, Department of Biological Sciences, College of Natural and Applied Sciences, Summit University Offa, Offa 4412, Kwara State, Nigeria; 4 Department of Physiology, School of Medical Sciences, Universiti Sains Malaysia, Kubang Kerian 16150, Kelantan, Malaysia; 5 Institute for Research in Molecular Medicine (INFORMM), Universiti Sains Malaysia, Kubang Kerian 11800, Kelantan, Malaysia; 6 Department of Medicine, School of Medical Sciences, Universiti Sains Malaysia, Health Campus, Kubang Kerian 11800, Kelantan, Malaysia; 7 Hospital Universiti Sains Malaysia, Health Campus, Kubang Kerian 16150, Kelantan, Malaysia

**Keywords:** spontaneous bacterial peritonitis, hepatitis B virus, hepatitis C virus, liver cirrhosis, prevalence, and cancer

## Abstract

Background and Aim: Spontaneous bacterial peritonitis (SBP) is a common infection in liver cirrhosis. This systematic review and meta-analysis provide detailed information on the prevalence of SBP among hepatitis B virus (HBV) and hepatitis C virus (HCV)-related liver cirrhosis globally. Methods: A systematic search for articles describing the prevalence of SBP in HBV and HCV-related cirrhosis was conducted following the Preferred Reporting Items for Systematic Reviews and Meta-analysis (PRISMA) guidelines. Our search returned ten (10) eligible articles involving 1713 viral cirrhosis cases representing eight (8) countries. A meta-analysis was performed on our eligible studies using the random effect model. A protocol was registered with PROSPERO (CRD42022321790). Results: The pooled prevalence of SBP in HBV-associated cirrhosis had the highest estimate [8.0% (95% CI, 2.7–21.0%; *I*^2^ = 96.13%; *p* < 0.001)], followed by SBP in HCV-associated liver cirrhosis [4.0% (95% CI, 1.3%–11.5%; *I*^2^ = 88.99%; *p* < 0.001)]. China (61.8%, CI: 57.1–66.3%), the USA (50.0%, CI: 34.6–65.4%), and Holland (31.1%, CI: 21.6–42.5%) had the highest estimate for SBP in HBV associated liver cirrhosis, SBP in HCV associated liver cirrhosis and SBP in HBV + HCV associated liver cirrhosis respectively. There was a significant difference in the prevalence of SBP in viral hepatitis-associated liver cirrhosis with the year of sampling and method of SBP detection at *P* < 0.001. There was an increase in SBP incidence at the beginning of 2016 across the liver cirrhosis in this study. Conclusion: The findings of this review revealed a rise in the incidence of SBP in viral hepatitis over the last decade. The latter indicates a possible future rise in the global prevalence of SBP among HBV and HCV-related liver cirrhosis.

## 1. Introduction

Spontaneous bacterial peritonitis (SBP) is a bacterial infection of ascites and a common complication in patients with cirrhosis, accounting for around 10–40% of all hospitalized cases with ascites and liver cirrhosis globally [1,2,3]. Spontaneous bacterial peritonitis has many predisposing factors, including lifestyle and disease conditions [4].

Liver cirrhosis, in a more histological concept, is a fibrosis-characterized diffusion of standard hepatocyte architecture into a structurally abnormal nodule [4] that reduces the functional mass index of the hepatocyte as well as its vascular architecture [5,6].

There are two aetiological primary evolutionary sources of cirrhosis, alcoholic liver cirrhosis, and nonalcoholic liver cirrhosis [4]. The latter is usually ascribed to viral aetiology. Nonalcoholic cirrhosis of the liver has been firmly associated with hepatitis B virus (HBV) and hepatitis C virus (HCV), or a coinfection of both (HBV + HCV) [7,8]. 

The infection from SBP can spread to other organs, causing more severe multi-organ failure with poor patient prognosis [7,9]. The incidence of SBP in liver cirrhosis differs with regions globally, and chronic Hepatitis B is the most common etiological condition of liver cirrhosis in sub-Saharan Africa and some parts of Asia [4].

The clinical pattern, pathophysiology, and natural history of SBP among viral hepatitis are still unclear. Still, there are theories that SBP in liver cirrhosis is likely due to translocation and overgrowth of intestinal bacteria, which is an integral step in the colonization and pathogenic stage of SBP infection [2].

The European Association for the Study of Liver (EASL) has established a preventative treatment for SBP in cirrhosis based on therapeutic studies. It is essential to distinguish between the two types of infections (nosocomial and community-acquired) because the source of SBP has a significant impact on the patient’s clinical outcomes [7]. 

The treatment of SBP in viral hepatitis depends on the causative hepatitis virus [6,7]. The link between the severity of hepatitis-related viral cirrhosis and genotypes and sub-genotypes has been documented [10]. 

A recent meta-analysis of global epidemiological significance revealed that the occurrence of SBP in liver cirrhosis was significant. Though this study has a good sample size, it only examined SBP in liver cirrhosis without a clear distinction between cirrhosis of alcoholic and nonalcoholic aetiology [11]. 

Although the epidemiological burden, outcome, and related morbidity of SBP in viral cirrhosis are increasing, there is still a need for more information on the pooled prevalence analysis of SBP in patients with HBV- and HCV-related cirrhosis. Thus, this systematic literature review and meta-analysis were conducted to determine the pooled prevalence of SBP in patients with viral-related cirrhosis and to investigate the importance of individual hepatitis cirrhosis-causing viruses. To the best of our knowledge, this study is the first systematic review and meta-analysis reporting the prevalence of SBP in viral cirrhosis, the findings of this study will provide baseline data on the epidemiology, trend, and potential pattern of SBP endemicity globally. It will also help healthcare givers diagnose, treat, and manage SBP in viral-cirrhotic patients.

## 2. Methods

A study protocol was lodged for this study with PROSPERO, an online systematic review database under the ID record number: CRD42022321790.

### 2.1. Study Design and Protocol

The Preferred Reporting Items for Systematic reviews and Metanalysis protocol guidelines were used as this study’s checklist (Supplementary File S1) [12]. 

### 2.2. Literature Review Search Strategy

The PROSPERO and Database of abstracts of review of effects (DARE) were searched to avoid duplication of an ongoing or existing review on our study topic: “Spontaneous bacterial peritonitis in HBV, HCV-based liver cirrhosis”. We searched four other international databases (PubMed, Scopus, Google Scholar, and ScienceDirect) on the occurrence of SBP in viral hepatitis-based cirrhosis after our preliminary search confirmed that there was neither an ongoing study nor an existing review on the topic of choice. The PubMed database was searched using standard search terms that best represented the study using the search strategy (“Spontaneous bacterial peritonitis” [All Fields] OR “Ascitic fluid infection” [All Fields] OR (“ascites” [All Fields] OR “ascites” [MeSH Terms] OR “ascites” [All Fields] OR “ascitic” [All Fields]) AND (“liver cirrhosis” [All Fields] OR “viral liver cirrhosis” [All Fields]) AND (“hepatitis B virus” [All Fields] AND (“hepatitis C virus” [All Fields] OR “HBV” [All Fields] OR “Hepatitis B” [All Fields]) OR “HCV” [All Fields] OR “Hepatitis C” [All Fields]). Details of the search strategy are found in Supplementary File S2.

The keyword search terms used for the comprehensive search in other databases aside from PubMed were “Spontaneous bacterial peritonitis”, “cirrhosis”, and “hepatitis”. The titles and references from the articles that met our inclusion criteria were utilized as an additional search tool. All searches were done without language restriction or year of study, and a final search was done on 5 November 2021. Two authors carried out an independent probe of the literature to reduce the chances of bias. All included literature references were imported into the Mendeley Reference manager to remove duplicates before screening the titles and abstracts. 

### 2.3. Inclusion and Exclusion Criteria

Studies with designs including retrospective studies, cross-sectional studies, prospective studies, case-control studies, and randomized clinical trials were included. Only original research that reported SBP in viral-related cirrhosis was included in this study. We excluded all reviews, short communications, commentaries, editorials, studies that reported SBP in alcoholic cirrhosis, studies that did not report SBP in viral-based cirrhosis, studies that reported ambiguous SBP information contained duplicated data, and studies that reported SBP in viral-based cirrhosis. Still, without clarity of the number of examined population, all studies with insufficient information (no clear description of the detection method, type of sample, period of sampling, viral cirrhosis record, country of study) and studies whose full text could not be retrieved.

### 2.4. Data Extraction

Two authors independently screened the title, abstract and full-text review of the recruited studies to extract all necessary information into a table. A third author reviewed the results, and all discrepancies between the authors were resolved by consensus. All literature was screened in three phases: title, abstract and full text. Information including the first author’s name, year of publication, country of publication, sample size, number of cases, type of viral hepatitis-based cirrhosis, and detection method were all extracted from each included study. Studies where viral cirrhosis was not defined, based on their aetiological agent, were categorized as both hepatitis B virus and hepatitis C virus-related cirrhosis.

### 2.5. Quality Assessment

The quality of all the studies was assessed using the Joanna Briggs Institute (JBI) prevalence data appraisal checklist (Supplementary File S3) independently by two authors [13]

The appraisal set nine (9) parameters that any standard research should meet. A scoring coding system of “zero (0) for NO” and “One (1) for YES” were assigned. The included studies were scored from 0-9 based on Joanna Brigg’s Institute critical appraisal for prevalence data. Studies with an overall score of less than seven (7) were considered unsuitable for this study. Studies with a quality score higher than seven were regarded as good quality for this study and were included. Studies with a quality score greater than seven were considered to be of good quality for the study. The quality score assessment for all the studies is provided in Supplementary File S4. Two authors carried out the quality assessment independently. All studies included in this study were recruited based on consensus between the reviewers.

### 2.6. Data Analysis

OpenMeta Analyst version 3.1 (CEBM, 2022, Providence, RI, USA) and Comprehensive Meta-Analysis software (Biostat.inc, 2021, Englewood, NJ, USA)) were used to analyze the data [14].

The pooled prevalence of SBP in viral hepatitis cirrhosis was calculated, and subgroup analyses were carried out according to the aetiological source of viral cirrhosis, year of sample collection, and region of study. Due to high variability in the sample collection and the period of sample collection, and the variation in the detection method, the random effect model best suits the study [15]. The DerSimonian and Laird method was used for the pool prevalence determination in this meta-analysis [16].

A forest plot was constructed to know the estimated weight of each study, its effect sizes, the proportional prevalence, the confidence interval of the estimated prevalence and the degree of heterogenicity [15]. Then, all data were transformed using logit transformation.

### 2.7. Heterogeneity Analysis and Publication Bias Test

Publication bias was examined using Egger’s regression test and funnel plots. The heterogeneity of the study level estimates was measured using the Inconsistency index Statistics (*I*^2^) OF > 75%, 50%, and 25%, which is interpreted as high, moderate and low heterogeneity. Cochrane Q test was also used to evaluate the heterogeneity [15]. Non-significant heterogeneity will be accepted if the ratio of Q and degree of freedom (df) is less than one (1) [Q: df < 1]. A subgroup meta-analysis was carried out to determine the sources of heterogeneity. The overall effect of each study on the pool prevalence and outcomes and in between study source of heterogeneity was examined using the “Leave One Out” meta-analysis sensitivity test. 

## 3. Results

### 3.1. Search Results and Eligible Studies

**Figure 1:**
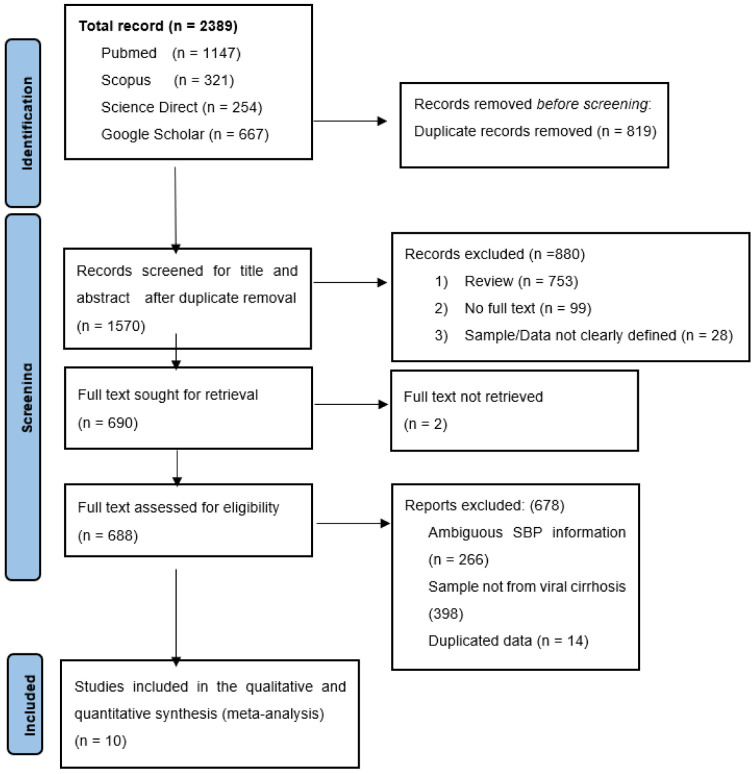
Summary of the article selection process.

Our comprehensive systematic search retrieved an initial 2389 abstracts from four databases. All duplicates were removed, and 819 articles were discarded based on their titles and abstracts. A total of 688 papers were eligible for full-text evaluation, but 678 studies were rejected as these did not meet our inclusion criteria or had a low JBI assessment score. Figure 1 shows a complete overview of the selection procedure. This systematic literature review and meta-analysis included ten (10) publications consisting of 1713 viral cirrhosis cases.

### 3.2. Characteristics of the Eligible Studies

The articles included in this study were conducted across six countries representing four continents. We recovered ten articles that met our inclusion criteria (Table 1) in eight countries. The United States of America (USA) and South Korea contributed 40% (*n* = 4) of the included articles. 

A total of 1713 viral cirrhosis was reported across ten (10) studies ranging from 675 viral cirrhosis (Pakistan) to 38 viral cirrhosis (USA) (Table 1). 

Three viral cirrhosis conditions with SBP were examined: (1) SBP among HBV cirrhosis; (2) SBP among HCV cirrhosis; and (3) SBP among HBV and HCV cirrhosis (Table 1). 

The most prominent method of SBP detection was culturing. Over 80% of the included were retrospective studies. Studies whose samples were collected from 2006 to 2015 (*n* = 4) and up to 2005 (*n* = 4) were the most dominant. Only 20% of the articles collected their samples from 2016 and above (*n* = 2) (Table 1).

A summary of included articles is provided in Table 1. The quality assessment shows that the majority of the included articles are of high methodological quality (Supplementary File S4). 

### 3.3. Prevalence of SBP in Viral Hepatitis-Related Cirrhosis 

**Figure 2:**
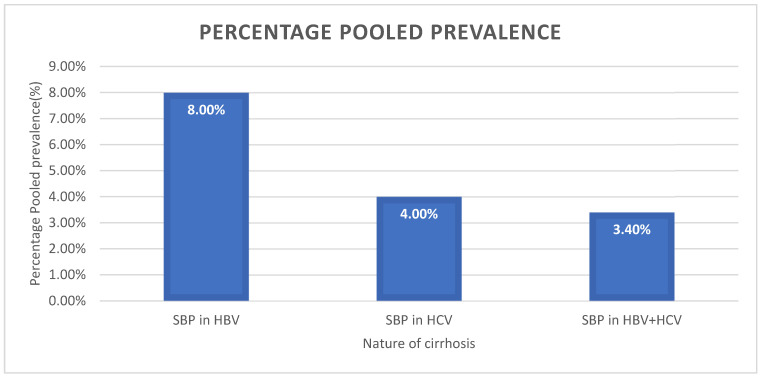
A bar chart showing the pooled prevalence of SBP in HBV and HCV cirrhosis.

The pooled prevalence of SBP in viral cirrhosis was diverse relative to the etiological agent of liver cirrhosis. There was a high significance level of SBP with all the viral categories responsible for liver cirrhosis. The pooled estimates of SBP across the three different viral cirrhosis categories (HBV, HCV, and HBV + HCV) were highly heterogenous, as the heterogeneity index for all the individual categories was greater than 75% (Figure 2).

**Figure 3:**
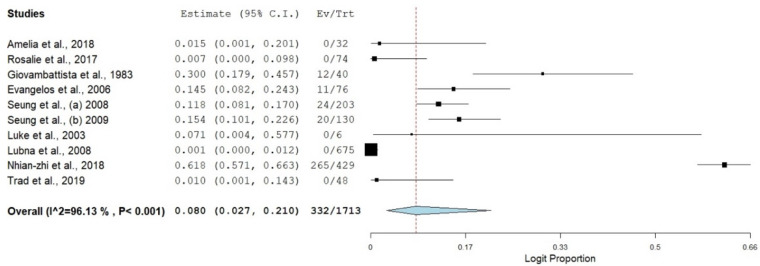
Forest plot showing SBP in HBV cirrhosis [17,18,19,20,21,22,23,24,25,26].

**Figure 4:**
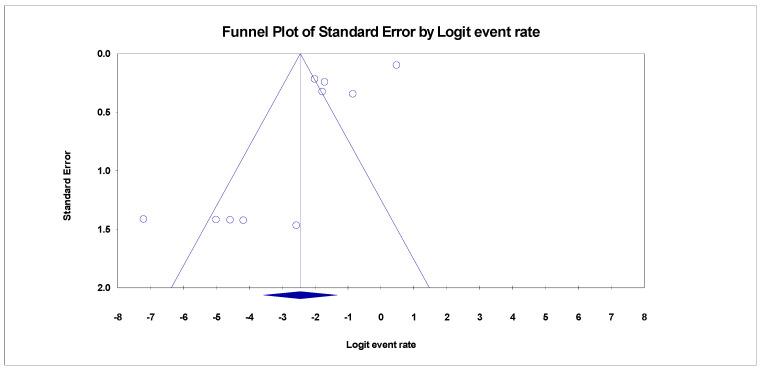
Funnel plot showing publication bias for SBP in HBV cirrhosis (Egger’s *p* = 0.01273).

**Figure 5:**
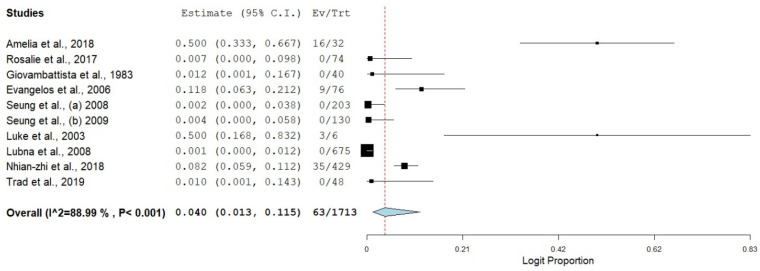
Forest plot showing SBP in HCV cirrhosis [17,18,19,20,21,22,23,24,25,26].

**Figure 6:**
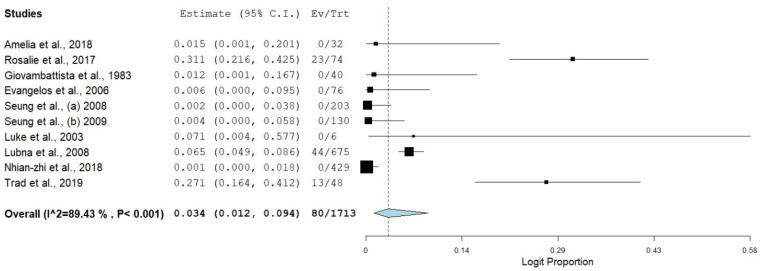
Forest plot showing SBP in HBV and HCV co-infected cirrhosis [17,18,19,20,21,22,23,24,25,26].

The pooled prevalence of SBP in HBV liver cirrhosis had the highest estimate [8.0% (95%CI: 2.7–21.0%, *I*^2^ = 96.13%, *p* ≤ 0.001)] (Figure 3 and Figure 4), followed by SBP in HCV associated liver cirrhosis, with an estimate of 4.0% (95%CI: 1.3–11.5%, *I*^2^ = 88.99%, *p* ≤ 0.001) (Figure 5). The pooled estimate of SBP in liver cirrhosis caused by both combination of HBV and HCV had the lowest prevalence in the category of SBP in viral cirrhosis [3.4% (95%CI: 1.2–9.4%, *I*^2^ = 89.43%, *p* ≤ 0.001)] (Figure 6).

### 3.4. Publication Bias

**Figure 7:**
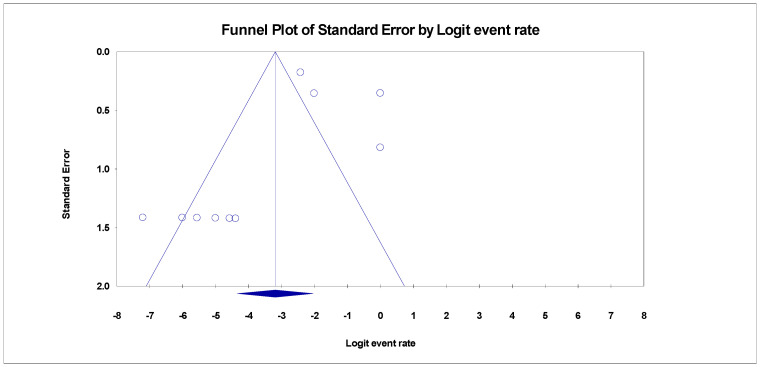
Funnel plot showing publication bias for SBP in HCV cirrhosis (Egger’s *p* = 0.28126).

Despite the diversity in the included studies for this review, there were pieces of evidence of publication bias in the funnel plots for the three categories. The adjusted trim and fill Duval, and Tweedies’s test for studies reporting SBP in HBV liver cirrhosis revealed four (4) adjusted trimmed studies with a Q-value of 253.55 in the random effect model adapted to the right (Table 2). Kendall’s Tau’s statistics revealed that the bias in the publication was not significant with and without continuity correction (*p* > 0.001) (Table 2). The prevalence of SBP in HCV liver cirrhosis also revealed evidence of publication bias (Figure 7). After adjusting the included studies with Duval and Tweedie’s trim and fill test using the random effect model, it was evident that four (4) studies were trimmed to the right with an adjusted Q value of 119.08. there was no significance in the correlation between the observed and the adjusted values as Kendall’s Tau’s *p*-value with and without continuity of correction were greater than 0.001 (Table 2). 

**Figure 8:**
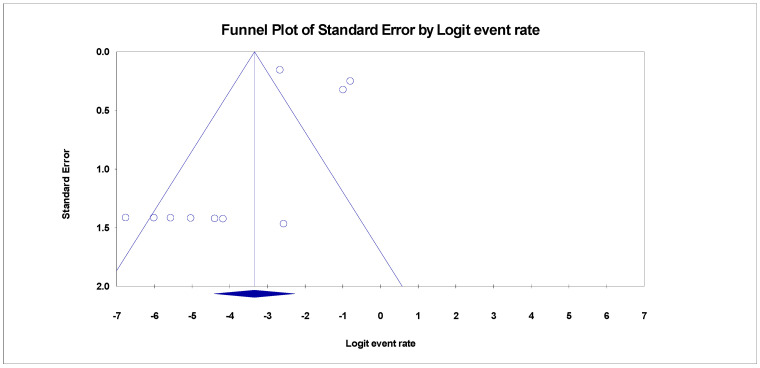
Funnel plot showing publication bias for SBP in HBC cirrhosis (Egger’s *p* = 0.24663).

The pooled estimate of SBP in a combination of HBV and HCV liver cirrhosis also showed publication bias. After Egger’s regression intercept was plotted (Figure 8), there was no significance in SBP pooled prevalence to the source of publication bias (Egger’s *p* = 0.247). After Duval and Tweedie’s trim and fill test, the adjusted Q value was 111.995. the trim and fill test revealed that four (4) studies were trimmed to the right in the random effect model. The Begg and Mazumdar rank correlation indicated that the Kendal Tau value was not significant with and without continuity of correction (*p* > 0.001) (Table 2). 

### 3.5. Subgroup Meta-Analysis

A subgroup analysis based on the country of study, year of study sampling, method of SBP detection, and the type of study designs was carried out to determine the sources of heterogeneity from the pooled studies, as substantial heterogeneity was observed. The overall subgroup analysis result based on the study’s country revealed a high degree of variability among studies reporting SBP in hepatitis liver cirrhosis. Studies reporting SBP in HBV cirrhosis across the eight countries had the overall Higgin *I*^2^ statistics of 96.13% and a heterogeneity Chi-square (Q) of 232.86 at *p* < 0.001. However, most studies were reportedly from the USA and Republic of Korea (n = 2 each). Similarly, studies reporting SBP in HCV cirrhosis had no significant Q and Higgin’s *I*^2^ index. Despite the latter, the overall pooled estimates revealed a high level of heterogeneity (*I*^2^ = 88.99%) and a significant Chi-square (81.73) at *p* < 0.001 (Table 3).

In comparison, studies reporting SBP in HBV + HCV liver cirrhosis also had a diverse level of heterogeneity within the countries with the overall Higgin’s *I^2^* statistics of 89.43% and a significant heterogeneity Chi-square value (65.12) at *p* < 0.001 (Table 3). 

The result of subgroup analysis by year of sampling revealed a very high level of heterogeneity among the years of sampling in the studies reporting SBP in hepatitis viral cirrhosis (Table 4). The overall heterogeneity index for studies reporting SBP in HBV cirrhosis was 96.13% with a significant Chi-square value (232.86) at *p* < 0.001. Studies whose samples were collected between the year 2006–2015 and studies whose samples were collected up to the year 2006 had the highest number of studies (n = 4 each) and were diversely heterogenous (*I*^2^ ≥ 75%). Studies whose samples were collected later than the year 2015 revealed no level of heterogeneity (*I^2^ =* 0.00%) and were also not significant (*p* = 0.842) (Table 4). 

Studies reporting SBP in HCV liver cirrhosis were highly heterogenous within groups based on their year of sampling (*I^2^* ≥ 75%). The overall Higgin’s *I*^2^ statistic revealed high variability in the studies reporting SBP in HCV liver cirrhosis (*I*^2^ *=* 88.99%) with a heterogeneity chi-square of 81.73 at *p* < 0.001. Studies whose samples were collected later than 2015 revealed the highest variability among the set categories of sampling periods in correlation to SBP in HCV liver cirrhosis (*I*^2^ *=* 89.75%). The heterogeneity index for studies reporting SBP in both HBV+HCV was diverse within the different years of sampling and was, therefore, highly heterogenous (*I*^2^ *=* >75%). Studies whose samples were collected between 2006–2015 revealed the highest level of heterogeneity (*I*^2^ *=* 82.87%). Though the studies whose samples were collected later than 2015 were heterogeneous (*I*^2^ *=* 78.93%), they were not statistically significant (*p* = 0.029) (Table 4).

Most of the studies included in this review were retrospective study designs (80% of the included studies). Retrospective study designs were statistically significant in all the categories. Study designs with SBP in only HBV cirrhosis revealed that retrospective studies had the highest estimate (9.2%) compared to case-control (1.5%) (Table 5). The overall pool heterogeneity index was greater than 75% for SBP in HBV cirrhosis. Retrospective studies were highly heterogeneous in distribution (96.87%) with a Q statistic of 223.41. Prospective and case-control designs had the highest estimate (50.0% each) for SBP in HCV cirrhosis. Prospective study designs had the highest estimate of SBP in both HBV and HCV cirrhosis (Table 5).

The two methods of detection were diversely heterogeneous across the three different categories. Culture detection had the highest estimate for SBP in HCV cirrhosis. The heterogeneity index for cell count and culture was moderate (62.7%) at a Q statistic of 5.32 and was not statistically significant. There was a high level of diversity in the studies reporting SBP in both HBV and HCV and studies reporting SBP in HCV (*I^2^* ≥ 80%) (Table 6).

## 4. Discussion 

This systematic review and meta-analysis aimed to determine the global prevalence of SBP in HBV and HCV liver cirrhosis. The findings of this review were based on the data collected from three categories of viral hepatitis cirrhosis (liver cirrhosis caused by HBV, liver cirrhosis caused by HCV, and liver cirrhosis caused by both HBV and HCV) globally. 

SBP in HBV liver cirrhosis had the highest prevalence (8.0%). The latter could be due to the relative immunological and epidemiological significance of HBV infection among patients [27]. Our findings are consistent with Smith et al. [28], who reported an HBV prevalence of >13.0%.

SBP coinfecting HBV+HCV-based cirrhosis had the lowest estimate. The latter could be attributed to several factors; the occurrence of HBV + HCV coinfection incidence is more common compared to other prevailing disease conditions/complications (SBP in HBV + HCV) associated with hepatitis and its progression into liver cirrhosis [29]. The immunological imprints and cell memories trigger during the viral clearance stage of hepatitis progression into liver cirrhosis are adequate to neutralize SBP infection [30]. 

The review included ten studies across four continents that met our inclusion criteria. Most of the studies were from the USA and South Korea, accounting for 40% of the included studies. The incidence of SBP in viral cirrhosis was significant in the USA and South Korea, despite the commendable hepatitis surveillance, prevention, and treatment schemes available in both nations

Country subgroup analysis revealed that China (61.8%) had the highest estimate of SBP in HBV liver cirrhosis. The latter could be due to the country’s high nosocomial and community-based SBP infection [31]. The findings of this study complement the report of Shi et al. [32], who reported a very high prevalence of SBP (87.4%) in China.

Italy also had a significant SBP in HBV cirrhosis (30.0%). The probable reason for the high prevalence is unclear. Still, it could be due to the high HBV prevalence in Italy despite the preventive measures already in place to curb the disease’s endemicity. The findings of this report agree with the description of Piano et al. [33], who reported a significant HBV occurrence in an Italian city despite the implementation of the HBV vaccination scheme.

The USA had a relatively low estimate of SBP in HBV cirrhosis in this study (3.3%). It can be attributed to the evolutionary shift of the HBV infection, as the overall prevalence of HBV infection in the USA has reduced drastically over the past two decades [34]. 

Despite the variation in the estimate of SBP in HBV cirrhosis in Asia (South Korea [13.3%], China [61.8%]), Pakistan had the lowest estimate (0.1%) in our study. The latter could be associated with the low morbidity of SBP in liver cirrhosis patients in Pakistan and can also be attributed to inadequate SBP reporting and documentation [35].

Tunisia had a low SBP in HBV cirrhosis estimate (1.0%). The latter could be due to the low estimate of SBP in North Africa and underreporting [36]. The burden of SBP in HCV liver cirrhosis had the highest estimate in the USA (50.0%). The latter may be due to the distribution of HCV infection in the USA and high-risk behaviours [37]. Our findings are consistent with the report of others [38,39].

The trend of SBP infection in HCV liver cirrhosis in Europe was diverse in our study, Greece had the highest estimate (11.8%), and Holland had the lowest estimate (0.7%). The latter could be due to the distribution pattern of SBP and HCV across Europe. This finding compliments the report of [40,41], who reported a variation in the distribution pattern of HCV in European countries.

Despite the high prevalence of SBP in HCV liver cirrhosis in China (8.2%), the trend of SBP prevalence in HCV cirrhosis in Pakistan (0.1%) and South Korea (0.3%) was relatively low. The latter could be attributed to the heavy epidemiological burden of HCV in China and the variation in the geographical distribution of HCV in Asia [42,43]. 

The prevalence of SBP in both HBV and HCV cirrhosis had the highest estimate in Holland (31.3%). The high estimate is not apparent, but it could be due to the immunological significance of HBV and HVC coinfection. The findings of this study correspond with other reports [30,44].

Most studies included in this systematic review and meta-analysis were carried out between the year >2006 to 2015, accounting for 80% of the study in this review. Studies whose samples were collected in years < 2006 had the highest estimate of SBP in HBV liver cirrhosis (12.9%), followed by studies whose samples were collected between the years 2006–2015 (11.0%). The burden of SBP in HBV liver cirrhosis reduced drastically towards the start of the year 2016 (1.2%). The probable reason for the sudden fall in the prevalence of SBP in HBV cirrhosis could be attributed to the improvement in the global health and care system and the impact of HBV vaccination/prevention programs globally [43]. 

The burden of SBP in HCV cirrhosis differs from SBP in HBV cirrhosis. There was a spike in the estimate of SBP in HCV-related cirrhosis at the start of 2016 (11.1%). Despite the reduction in the morbidity of SBP burden in HCV cirrhosis from year < 2006 (7.6%) to the duration between 2015–2015 (0.6%).

There was a sudden high trend of SBP prevalence among HCV cirrhosis at the start of 2016. The reason for the sudden surge in the prevalence of SBP in HCV cirrhosis is not apparent. Still, it could be attributed to several factors: lack of preventive vaccines for HCV, changes in the epidemiological spectra of the disease and the high level of degeneracy and diversification of the HCV progeny [38,45,46].

The prevalence of SBP in both HBV and HCV liver cirrhosis reduced in the year 2006–2015 (0.7%) after the initial high prevalence in year < 2006 (4.9), this could be attributed to the effectiveness of the HBV preventive vaccine and adequate awareness of the disease by the appropriate agencies [47]. 

Despite the drop in SBP incidence among HBV + HCV cases between 2006–2015, there was a rise in the overall prevalence of SBP in HBV + HCV cirrhosis in year > 2016 (9.3%). The probable reason for the spike in SBP cases in HBV + HCV could be attributed to patients’ low immunity because of dual HBV and HCV infection [29].

There were three study designs for our study: case-control, retrospective and prospective study design. Eighty percent (80%) of the included research were retrospective studies. The latter could be due to the overall burden of SBP in liver cirrhosis over a long period [11]. In comparison, the retrospective study design had the highest estimate of SBP in HBV cirrhosis (9.2%), while case-control studies had the highest estimate of SBP in HCV liver cirrhosis (50.0%). Studies reporting prospective design had the highest estimate of SBP in both HBV and HCV cirrhosis (7.1%). The variation in the estimate of SBP in the three different Hepatitis cirrhosis cases in our review could be due to other study designs. These findings disagree with the report of Kim et al. [48], who reported that differences in study designs do not significantly impact the prevalence of a disease. 

A subgroup meta-analysis was carried out on the method of SBP detection in viral hepatitis liver cirrhosis. Our findings revealed that most of our studies (70%) reported only the cultural method as the mean of SBP detection. In comparison, others presented cell counting and culturing (30%) as their means of SBP detection. The burden of SBP in studies reporting culturing was high across different groups. Comparatively, SBP in HBV cirrhosis and SBP in HCV cirrhosis had their highest estimate in studies reporting culturing as means of SBP detection (HBV cirrhosis 10.7% and HCV cirrhosis 4.1%). There was an equivocal estimate of SBP in HBV+HCV cirrhosis (2.9%), and there was no significant change in the prevalence of SBP in the examined detection method. The latter could be due to the preference for culturing over other methods in SBP detection [4]. This systematic review and meta-analysis have its strength and limitations. The strength includes the thorough analysis of the available data across four continents. It is the first systematic review and meta-analysis of SBP in viral hepatitis-associated liver cirrhosis. The study’s main limitation is the low data pool from some countries. The latter could give a misleading pool prevalence of the actual incidence of SBP in viral hepatitis-related cirrhosis. 

## 5. Conclusions

This systematic review and meta-analysis of SBP in viral hepatitis-associated liver cirrhosis reveal a high prevalence of SBP in HBV-related cirrhosis (8.0%) and SBP in HCV-related cirrhosis (4.0%), there was an uprise in the occurrence of SBP in viral hepatitis over the last decade. The latter indicates a possible hike in the global prevalence of SBP among viral hepatitis-associated liver cirrhosis. This review will help governments and governmental agencies to set policies toward curbing the morbidity of SBP in viral hepatitis liver cirrhosis globally.

## Figures and Tables

**Table 1:** Characteristics of the included studies showing the occurrence of SBP in HBV and HCV-related liver cirrhosis globally.

Author, Year	Year	Country	Sampling Period	HBV Sampled	SBP in HBV	HCV Sampled	SBP in HCV	HBV + HCV Sampled	SBP in HBV + HCV	Study Design	Method of Detection
Amelia et al. [17]	2018	USA	November 2011–March 2016	0	0	32	16	0	0	Case-control	Cell count and culture
Rosalie et al. [18]	2017	Holland	January 2003–December 2005	0	0	0	0	74	23	Retrospective	Culture
Giovambattista et al. [19]	1983	Italy	December 1976–December 1978	40	12	0	0	0	0	Retrospective	Culture
Evangelos et al. [20]	2006	Greece	June 1999–June 2001	49	11	27	9	0	0	Retrospective	Culture
Seung et al. (a) [21]	2008	South Korea	January 1996–December 2015	203	24	0	0	0	0	Retrospective	Cell count and culture
Seung et al. (b) [22]	2009	South Korea	January 1998–December 2007	130	20	0	0	0	0	Retrospective	Culture
Luke et al. [23]	2003	USA	July 1994–December 2002	0	0	6	3	0	0	Prospective	Culture
Lubna et al. [24]	2008	Pakistan	Nov 2005–December 2007	0	0	0	0	675	44	Retrospective	Culture
Nhian-Zhi et al., [25]	2018	China	January 2012–December 2015	370	265	59	35	0	0	Retrospective	Culture
Trad et al. [26]	2019	Tunisia	January 2003–December 2017	0	0	0	0	48	13	Retrospective	Cell count and culture

**Table 2:** Tau statistic and point estimate.

	Random Effect Model				
				Kendal Tau’s *p*	
	Studies Trimmed	Point Estimates	Q Value	Without Continuity Correction	With Continuity Correction
SBP in HBV cirrhosis					
Adjusted	4	1.299	253.55	0.180	0.211
Observed	-	2.448	232.86		
SBP in HCV cirrhosis					
Adjusted	4	1.916	119.08	0.788	0.858
Observed	-	3.189	81.73		
SBP in HBV + HCV cirrhosis					
Adjusted	4	2.213	112.00	0.180	0.211
Observed	-	3.342	85.12		

**Table 3:** Subgroup analysis of SBP in three different viral hepatitis-related liver cirrhosis across countries.

Country	Number of Studies	Prevalence (%)	95% CI	*I*^2^ (%)	Q	Heterogeneity Test	
						DF	*P*
SBP in both HBV and HCV cirrhosis							
USA	2	3.3	0.5–19.9	0.00	0.619	1	0.431
Holland	1	31.1	21.6–42.5	-	-	-	-
Italy	1	1.2	0.1–16.7	-	-	-	-
Greece	1	0.6	0.0–9.5	-	-	-	-
South Korea	2	0.3	0.0–2.1	0.00	0.049	1	0.824
Pakistan	1	0.1	4.9–8.6	-	-	-	-
China	1	0.1	0.0–1.8	-	-	-	-
Tunisia	1	27.1	16.4–32.5	-	-	-	-
Overall	10	3.4	1.2–9.4	89.43	65.12	9	<0.001
SBP in HCV cirrhosis							
USA	2	50.0	34.6–65.4	0.00	0.00	1	1.000
Holland	1	0.7	0.0–9.8	-	-	-	-
Italy	1	1.2	0.1–16.7	-	-	-	-
Greece	1	11.8	6.3–21.2	-	-	-	-
South Korea	2	0.3	0.0–2.1	0.00	0.049	1	0.824
Pakistan	1	0.1	0.0–1.2	-	-	-	-
China	1	8.2	5.9–11.2	-	-	-	-
Tunisia	1	1.0	0.1–14.3	-	-	-	-
Overall	10	4.0	1.3–11.5	88.99	81.73	9	<0.001
SBP in HBV cirrhosis							
USA	2	3.3	0.5–19.9	0.00	0.619	1	0.431
Holland	1	0.7	0.0–9.8	-	-	-	-
Italy	1	30.0	17.9–45.7	-	-	-	-
Greece	1	14.5	8.2–24.3	-	-	-	-
South Korea	2	13.3	10.1–17.4	0.00	0.872	1	0.350
Pakistan	1	0.1	0.0–1.2	-	-	-	-
China	1	61.8	57.1–66.3	-	-	-	-
Tunisia	1	1.0	0.1–14.3	-	-	-	-
Overall	10	8.0	2.7–21.0	96.13	232.86	9	<0.001

**Table 4:** Subgroup analysis of SBP in three different viral hepatitis-related liver cirrhosis with the year of sampling.

Sampling Period	Number of Studies	Prevalence (%)	95% CI	*I*^2^ (%)	Q	Heterogeneity Test	
						DF	*P*
SBP in HBV cirrhosis							
>2016	2	1.2	0.2–8.3	0.00	0.040	1	0.842
2006–2015	4	11.0	2.1–41.9	98.32	178.95	3	<0.001
<2006	4	12.9	4.6–31.7	72.48	10.90	3	0.012
Overall	10	8.0	2.7–21.0	96.13	232.86	9	<0.001
SBP in HCV cirrhosis							
>2016	2	11.1	0.1–91.6	89.75	9.75	1	0.002
2006–2015	4	0.6	0.0–8.7	86.25	21.81	3	<0.001
<2006	4	7.6	1.3–34.1	77.46	13.31	3	0.004
Overall	10	4.0	11.5–58.8	88.99	81.73	9	<0.001
SBP in both HBV and HCV cirrhosis							
>2016	2	9.3	0.5–68.6	78.93	4.75	1	0.029
2006–2015	4	0.7	0.1–7.3	82.87	17.52	3	<0.001
<2006	4	4.9	0.5–36.0	80.61	15.48	3	0.001
Overall	10	3.4	1.2–9.4	89.43	85.12	9	<0.001

**Table 5:** Subgroup analysis of SBP in three different viral hepatitis-related liver cirrhosis with the study design.

Study Design	Number of Studies	Prevalence (%)	95% CI	*I*^2^ (%)	Q	Heterogeneity Test	
						DF	*P*
SBP in HBV cirrhosis							
Case-control	1	1.5	0.1–20.1	-	-	-	-
Retrospective	8	9.2	3.0–25.3	96.87	223.41	7	<0.001
Prospective	1	7.1	0.4–57.7	-	-	-	-
Overall	10	8.0	2.7–21.0	96.13	232.86	9	<0.001
SBP in HCV cirrhosis							
Case-control	1	50.0	33.3–66.7	-	-	-	-
Retrospective	8	1.7	0.6–5.0	77.35	30.91	7	<0.001
Prospective	1	50.0	16.8–83.2	-	-	-	-
Overall	10	4.0	1.3–11.5	88.99	81.73	9	<0.001
SBP in both HBV and HCV cirrhosis							
Case-control	1	1.5	0.1–20.1	-	-	-	-
Retrospective	8	3.4	1.1–10.2	91.56	82.94	7	<0.001
Prospective	1	7.1	0.4–57.7	-	-	-	-
Overall	10	3.4	1.2–9.4	89.43	85.12	9	<0.001

**Table 6:** Subgroup analysis of SBP in three different viral hepatitis-related liver cirrhosis with the detection method.

Method of Detection	Number of Studies	Prevalence (%)	95% CI	*I*^2^ (%)	Q	Heterogeneity Test	
						DF	*P*
SBP in HBV cirrhosis							
Cell count and culture	3	4.1	0.6–21.7	62.4	5.32	2	0.007
Only culture	7	10.7	3.2–30.3	95.97	148.75	6	<0.001
Overall	8.0	2.7	2.7–57.2	96.13	232.86	9	<0.001
SBP in HCV cirrhosis							
Cell count and culture	3	3.3	0.0–70.8	92.09	25.29	2	<0.001
Only culture	7	4.1	1.4–11.6	81.1	31.75	6	<0.001
Overall	8.0	4.0	1.3–11.5	88.99	81.73	9	<0.001
SBP in both HBV and HCV cirrhosis							
Cell count and culture	3	2.9	0.1–46.4	87.48	15.97	2	<0.001
Only culture	7	2.9	0.8–10.2	90.37	62.28	6	<0.001
Overall	8.0	3.4	1.2–9.4	89.43	85.12	9	<0.001

## Data Availability

The data presented in this study are available in the supplementary material.

## Supplementary Materials

The following supporting information can be downloaded at: https://www.mdpi.com/article/10.3390/healthcare11020275/s1, File S1: PRISMA 2009 checklist. File S2: Search strategy. File S3: Joanna Briggs Institute (JBI) critical appraisal checklist for prevalence data. File S4: Quality assessment of the included studies.
