# Supplementary material for: Prevalence of Spontaneous Bacterial Peritonitis (SBP) in Hepatitis B (HBV), and Hepatitis C (HCV) Liver Cirrhosis: A Systematic Review and Meta-Analysis
Source: Healthcare (Basel). 2023 Jan 16;11(2):275. doi: 10.3390/healthcare11020275 (PMC9859562; doi:10.3390/healthcare11020275)
Supplement: Supplementary file 1 [file healthcare-11-00275-s001.zip › SUPPLIMENTARY FILE/File S2 SEARCH STRATEGY.pdf]

SEARCH STRATEGY : SBP IN HEPATITIS BASED CIRRHOSIS

SCOPUS

TITLE-ABS-KEY("Spontaneous bacterial peritonitis" OR "SBP") AND TITLE-ABS-KEY("hepatitis" OR "hepatitis B" OR "hepatitis C") AND TITLE-ABS-KEY(cirrhosis)

GOOGLE SCHOLAR

allintitle: spontaneous bacterial peritonitis Cirrhosis "Hepatitis "

SCIENCE DIRECT

("Spontaneous bacterial peritonitis" OR "SBP") AND ("hepatitis" OR "hepatitis B" OR "hepatitis C") AND (cirrhosis)

PUB MED:

("spontaneous bacterial infection"[Title/Abstract] OR "SBP"[Title/Abstract] OR "Ascites"[Title/Abstract] OR (("ascetic"[All Fields] OR "ascetics"[All Fields]) AND "fluid infection"[Title/Abstract])) AND ("hepatitis"[Title/Abstract] OR "hepatitis b"[Title/Abstract] OR "hepatitis c"[Title/Abstract] OR "HBV"[Title/Abstract] OR "HCV"[Title/Abstract]) AND ("liver cirrhosis"[Title/Abstract] OR "cirrhosis"[Title/Abstract])
