# Supplementary material for: Prevalence of Spontaneous Bacterial Peritonitis (SBP) in Hepatitis B (HBV), and Hepatitis C (HCV) Liver Cirrhosis: A Systematic Review and Meta-Analysis
Source: Healthcare (Basel). 2023 Jan 16;11(2):275. doi: 10.3390/healthcare11020275 (PMC9859562; doi:10.3390/healthcare11020275)
Supplement: Supplementary file 1 [file healthcare-11-00275-s001.zip › SUPPLIMENTARY FILE/File S4 QUALITY ASSESMENT OF THE STUDIES .pdf]

QUALITY OF INCLUDED STUDIES BY JBI CRITICAL APPRAISAL CHECKLIST FOR STUDIES REPORTING PREVALENCE DATA

S/N	Name of authors and year of publication		JBI checklist*									Total
1	Amelia et al.	2018	1	2	3	4	5	6	7	8	9	
2	Rosalie et al.	2017	Yes	No	Yes	Yes	Yes	Yes	Yes	Yes	Yes	8
3	Giovambattista et al.	1983	Yes	No	Yes	Yes	Yes	Yes	Yes	Yes	Yes	8
4	Evangelos et al.	2006	Yes	No	Yes	Yes	Yes	Yes	Yes	Yes	Yes	8
5	Seung et al., (a)	2008	Yes	No	Yes	Yes	Yes	Yes	Yes	Yes	Yes	8
6	Seung et al., (b)	2009	Yes	No	Yes	Yes	Yes	Yes	Yes	Yes	Yes	8
7	Luke et al.	2003	Yes	No	Yes	Yes	Yes	Yes	Yes	Yes	Yes	8
8	Lubna et al.	2008	Yes	No	Yes	Yes	Yes	Yes	Yes	Yes	Yes	8
9	Nhian-Zhi et al.	2018	Yes	No	Yes	Yes	Yes	Yes	Yes	Yes	Yes	8
10	Trad et al.	2019	Yes	No	Yes	Yes	Yes	Yes	Yes	Yes	Yes	8

JBI CHECKLIST* 1. Appropriate sampling frame to address target population, **2.** Appropriate sampling way of study participants, **3.** Adequate sample size, **4.** Detail description of study participants and settings, **5.** Data analysis with sufficient coverage of identified sample, **6.** Use of valid methods to identify the condition, **7.** Standard, reliable way of measurement of condition for all participants, **8.** Availability of appropriate statistical analysis, **9.** Adequate response rate and management of low response rate.

Scores are coded as Yes=1 and No=0.
